# ZEB1 confers stem cell-like properties in breast cancer by targeting neurogenin-3

**DOI:** 10.18632/oncotarget.17077

**Published:** 2017-04-13

**Authors:** Chen Zhou, Huimin Jiang, Zhen Zhang, Guomin Zhang, Hang Wang, Quansheng Zhang, Peiqing Sun, Rong Xiang, Shuang Yang

**Affiliations:** ^1^ Tianjin Key Laboratory of Tumor Microenvironment and Neurovascular Regulation, Medical School of Nankai University, Tianjin 300071, China; ^2^ Tianjin Key Laboratory of Organ Transplantation, Tianjin First Center Hospital, Tianjin 300192, China; ^3^ Department of Cancer Biology, Wake Forest University School of Medicine, Winston-Salem, NC 27157, USA

**Keywords:** breast cancer, neurogenin-3, stemness properties, tumor initiation, ZEB1

## Abstract

Cancer stem cells (CSCs) are a subpopulation of cancer cells believed to be implicated in cancer initiation, progression, and recurrence. Here, we report that ectopic expression of zinc finger E-box binding homeobox 1 protein (ZEB1) results in the acquisition of CSC properties by breast cancer cells, leading to tumor initiation and progression *in vitro* and *in vivo*. The neurogenin 3 gene (*Ngn3*) is a bona fide target of ZEB1, and its repression is a key factor contributing to ZEB1-induced cancer cell stemness. ZEB1 suppressed *Ngn3* transcription by forming a ZEB1/DNA methyltransferase (DNMT)3B/histone deacetylase 1 (HDAC1) complex on the *Ngn3* promoter, leading to promoter hypermethylation and gene silencing. The rescue of *Ngn3* expression attenuated ZEB1-induced cancer stemness and symmetric CSC division. Immunohistological analysis of human breast cancer specimens revealed a strong inverse relationship between ZEB1 and NGN3 protein expression. Thus, our findings suggest ZEB1-mediated silencing of *Ngn3* is required for breast tumor initiation and maintenance. Targeted therapies against the ZEB1/Ngn3 axis may be highly valuable for the prevention and treatment of breast cancer.

## INTRODUCTION

Although cancer stem cells (CSCs) constitute just a small fraction of the total tumor cell population, they are thought to be the main drivers of tumorigenesis and cancer recurrence [1, 2]. There is evidence that CSC numbers correlate well with aggressive tumor growth [3, 4]; moreover, specific changes in the microenvironment may induce, through mutagenic events or epigenetic alterations, a CSC expansion that potentiates tumor progression [5, 6]. Current knowledge indicates that CSC properties may be largely determined by evolutionarily conserved mechanisms dictating cell polarity and asymmetric cell division. Normal CSCs usually undergo asymmetric cell division, giving rise to daughter cells with different characteristics. Thus, this process results in simultaneous differentiation and self-renewal, while maintaining the number of stem cells constant for tissue homeostasis [7]. Symmetric cell division, in contrast, generates only stem cells. Whereas the resulting expansion of the stem cell population could be useful for tissue regeneration under normal physiological conditions, the deregulation of asymmetric and/or symmetric division may, under pathogenic conditions, promote tumor initiation [8]. For example, loss of tumor suppressor genes such as the adenomatous polyposis coli gene (APC), often favors increased symmetric division of CSCs, potentially leading to tumorigenesis [9]. Therefore, understanding the complex regulatory network that interconnects cell polarity and cell fate is paramount to elucidate the role of CSCs in carcinogenesis and to effectively guide preventive and therapeutic efforts.

Zinc finger E-box binding homeobox 1 (ZEB1), a pivotal member of the zinc finger-homeodomain transcription factor family, importantly influences developmental and homeostatic cell fate decision in a broad range of tissues [10–12]. The overexpression of ZEB1, demonstrated in breast, colon, prostate, and pancreatic cancer [13–16], contributes to impaired cell adherence and polarity [17], induction of epithelial to mesenchymal transition (EMT) [18], and the acquisition of chemo- and radio-resistant phenotypes [19, 20]. A growing body of evidence suggests that ZEB1 promotes the generation of breast CSCs [21–23]. For instance, elevated expression of ZEB1 predicts radiotherapy relapse in triple-negative breast cancer patients, in which the CSC population is enlarged [20]. Moreover, ectopic expression of ZEB1 in breast cancer is readily influenced by microenvironmental signals such as TGF-β, resulting in CSCs population expansion by non-CSCs to CSCs conversion and increased tumorigenesis [24]. Interestingly, ZEB1-related stemness properties have been shown to arise rather indirectly, through the transcriptional regulation of miRNAs such as miR-200 and miR-203 [18]. However, the cellular and molecular mechanisms downstream of ZEB1 that contribute to tumorigenesis remain largely obscure.

In this study, we provide evidence that ZEB1 promotes CSC self-renewal *in vitro* and *in vivo*, thus leading to increased breast cancer initiation and growth. By reduced representation bisulfite sequencing (RRBS) analysis, we identified neurogenin-3 (*Ngn3*) as a *bona fide* target of ZEB1 implicated in the acquisition of cancer cell stemness. ZEB1 represses *Ngn3* transcription by recruiting DNA methyltransferase (DNMT)3B and histone deacetylase (HDAC)1 to the *Ngn3* promoter, thus resulting in DNA hypermethylation and the silencing of *Ngn3*. We also demonstrated an inverse correlation between ZEB1 and NGN3 expression in human breast cancer specimens. Notably, rescuing *Ngn3* expression significantly attenuated ZEB1-induced CSC self-renewal and stemness. Our data elucidates an important role for ZEB1/*Ngn3* signaling in regulating stem cell fate and suggests a potential therapeutic target for ZEB1-overexpressing cancers.

## RESULTS

### ZEB1 induces stemness properties in breast cancer cells

**Figure 1:**
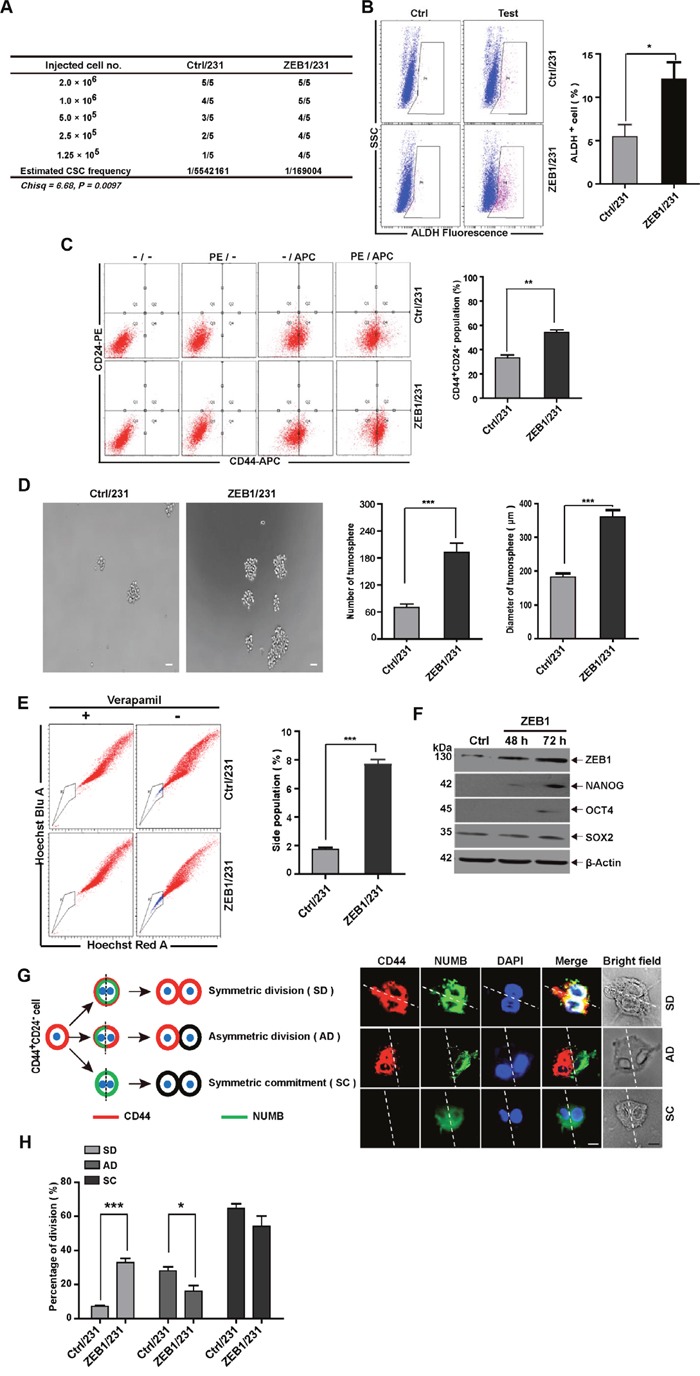
ZEB1 induces stemness properties in breast cancer cells **(A)** For *in vivo* limited dilution assays, a total of 2×10^6^, 1.0×10^6^, 5×10^5^, 2.5×10^5^, and 1.25×10^5^ ZEB1/231 or Ctrl/231 cells were injected into the mammary fat pads of nude mice. After 15 days, the mice were euthanized, and estimated CSC frequency was analyzed using ELDA software. Data were analyzed with chi-square tests (*P = 0.097). **(B)** and **(C)** Tumor tissues were prepared in single-cell suspension and processed for ALDH activity (B) and CD44^+^CD24^-^ population (C) analysis by flow cytometry. *P < 0.05, **P < 0.01 vs. the respective control by Student's *t*-test. **(D)** ZEB1/231 or Ctrl/231 cells were prepared in single-cell suspension and seeded in 24-well ultra-low attachment plates at 1,000 cells/well. Tumorsphere formation was examined after 14 days of culturing. ***P < 0.001 vs. the respective control by Student's *t*-test. **(E)** ZEB1/231 or Ctrl/231 cells were stained with Hoechst 33342 in the presence or absence of verapamil. The side population fraction was examined by flow cytometry. ***P < 0.001 vs. the respective control by Student's *t*-test. **(F)** The full-length ZEB1 expression plasmid was introduced into MDA-MB-231 cells. At the indicated time points, the expression of ZEB1, NANOG, OCT4, and SOX2 was assessed by immunoblotting and was normalized to the levels of β-Actin. **(G)** Schematic showing SD, AD, and SC patterns. **(H)** Confocal fluorescence images showing CD44 (red) and NUMB (green) intracellular distribution during the first cell division of the CD44^+^CD24^-^ population isolated from ZEB1/231 and Ctrl/231 cells and cultured in suspension with blebbistatin treatment. The percentage of the SD versus AD populations was analyzed. *P < 0.05, ***P < 0.001 vs. the respective control by Student's *t*-test. Scale bars: 20 μm.

To assess the influence of ZEB1 expression on breast cancer initiation *in vivo*, we performed extreme limiting dilution analysis to detect ZEB1-induced CSC frequencies. To this end, ZEB1 gain-of-function transfection was performed in MDA-MB-231 breast cancer cells to establish a nude mouse xenograft model (Supplementary Figure 1A). Compared with control (Ctrl/231) tumor cells, ZEB1/231 tumor cells displayed a 32.8-fold increase in CSC frequency (Figure 1A and Supplementary Figure 1B). Flow cytometry and immunohistochemical staining analysis further revealed increased activity of ALDH, a malignant human mammary stem cell marker [21], in tumors from ZEB1/231 xenografts (Figure 1B and Supplementary Figure 1C). In addition, measurement of the CD44^+^CD24^−^ breast CSC population also demonstrated that ectopic expression of ZEB1 led to increased stemness properties in xenografted MDA-MB-231 breast cancer cells (Figure 1C).

Next, by measuring three well-established CSC endpoints, i.e. tumorsphere formation, side population (SP) percentage, and stemness-related gene expression, we conducted *in vitro* studies to confirm that ZEB1 targets, in fact, breast CSCs. Our results demonstrated that ectopic ZEB1 expression in MDA-MB-231 cells increased tumorsphere formation (Figure 1D), SP percentage (Figure 1E), and the expression of pluripotency markers such as NANOG, OCT4, and SOX2 (Figure 1F).

Additionally, we explored the effect of ZEB1 on CSC polarity during division by assessing, shortly after mitosis, the subcellular distribution of NUMB, a protein involved in cell fate specification [25]. To this end, cell division symmetry was evaluated in ZEB1/231 and Ctrl/231 cells treated with blebbistatin, a cytokinesis-arresting small molecule that induces formation of binucleated cells (Figure 1G). Results showed that CD44 expression was abolished in up to 64.7% of mitotic Ctrl/231 cells, while NUMB was uniformly distributed around the actin cortex (Figure 1G and 1H), suggesting that both daughter cells lost their stem cell identity upon symmetric cell division (symmetric commitment [SC]). In 27.3% of the Ctrl/231 cells, CD44 and NUMB were asymmetrically distributed [asymmetric division [AD]), wherein NUMB was present only in CD44^-^ progeny, but not in CD44^+^, stem cell-like cells (Figure 1G and 1H). In up to 8.0% of the binucleated Ctrl/231 cells, on the other hand, CD44 expression was symmetric, and NUMB showed a uniform distribution, thus manifesting a symmetric division (SD) phenotype. Interestingly, as shown in Figure 1H, this SD phenotype was substantially enhanced after ectopic expression of ZEB1 (8.0% to 32.1%). In contrast, ZEB1 depletion achieved the opposite effect, i.e. reduced cancer stem-like cell properties (Supplementary Figure 2). These observations were not unique to MDA-MB-231 cells; ZEB1 overexpression or depletion in SUM-159 cells also resulted in enhanced and reduced CSC phenotypes, respectively (Supplementary Figure 3 and 4). In addition to the results from the previous study indicating that ZEB1 is capable of inducing EMT [26], the present data also suggest that ZEB1 is crucial for the acquisition or conservation of stemness properties in breast CSCs.

### ZEB1 downregulates Ngn3 via promoter hypermethylation

**Figure 2:**
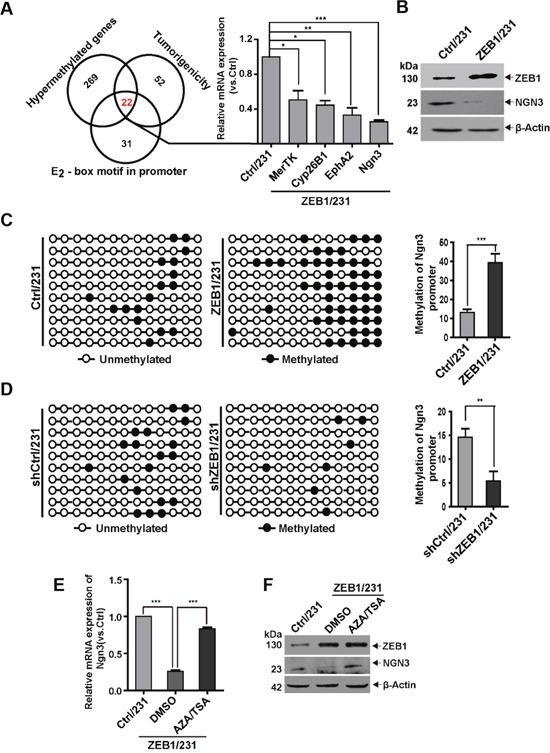
The *Ngn3* promoter is a *bona fide* hypermethylation target of ZEB1 **(A)** Schematic representation of RRBS identification of the putative ZEB1 targets involved in breast tumorigenesis. Quantitative PCR were performed to verify the downregulation of *MerTK*, *Cyp26B1*, *EphA2*, and *Ngn3* expression in ZEB1/231 vs. Ctrl/231 cells. *P < 0.05, **P < 0.01, ***P < 0.001 vs. the respective control by Student's *t*-test. **(B)** The downregulation of NGN3 protein expression was assessed by immunoblotting in ZEB1/231 vs. Ctrl/231 cells and normalized to the levels of β-Actin. **(C)** and **(D)** The percentage of DNA methylation of the *Ngn3* promoter was determined by BSP in ZEB1/231 vs. Ctrl/231 cells (C) and in shZEB1/231 vs. shCtrl/231 cells (D). **P < 0.01, ***P < 0.001 vs. the respective control by Student's *t*-test. **(E)** and **(F)** ZEB1/231 and Ctrl/231 cells were treated with AZA (1.5 μM) and TSA (2 mM) for the indicated time points. The expression of *Ngn3* was assessed by quantitative PCR (E) and immunoblotting (F) and normalized to the levels of β-Actin. ***P < 0.001 vs. the respective control by Student's *t*-test.

Based on mounting evidence implying a contribution of ZEB1 to epigenetic regulation during tumorigenesis [27], we performed RRBS to identify endogenous targets of ZEB1 in MDA-MB-231 cells. We identified 291 genes containing differentially methylated regions (DMRs) in ZEB1/231 cells, as compared with Ctrl/231 cells. 22 of these genes possess potential tumorigenic functions and contain E_2_-box motifs for ZEB1 binding in their promoter regions (Figure 2A). We then performed quantitative PCR to determine the correlation between ZEB1 and these differentially methylated genes in ZEB1/231 cells. The results showed that ectopic ZEB1 expression significantly downregulated the mRNA levels of *MerTK* (c-mer proto-oncogene tyrosine kinase), *Cyp26B1* (Cytochrome P450 26B1), *EphA2* (ephrin type-A receptor 2), and *Ngn3* (Figure 2A). Considering that *Ngn3* influences stem cell properties and cell differentiation [28], immunoblotting was performed to verify that a negative correlation existed between ZEB1 and NGN3 protein expression (Figure 2B). Thus, these data indicate that ZEB1 promotes CSC renewal through repression of *Ngn3* expression.

To further confirm that the inhibition of *Ngn3* expression by ZEB1 in breast cancer is correlated with DNA methylation, we conducted bisulfite sequencing PCR (BSP) to evaluate the methylation status of 13 CpG residues in the 243-bp DMR region (-164/+79) of the *Ngn3* promoter (Supplementary Figure 5A). Relative to Ctrl/231, DNA methylation was increased in ZEB1/231 cells (Figure 2C and Supplementary Figure 5B), while ZEB1 knockdown correlated instead with decreased DNA methylation (Figure 2D and Supplementary Figure 5C). *Ngn3* mRNA expression was then compared by quantitative PCR in Ctrl/231 vs. ZEB1/231 cells and in shCtrl/231 vs. shZEB1/231 cells (Supplementary Figure 5D and 5E), confirming a negative regulation of *Ngn3* expression by ZEB1.

Gene regulation via promoter methylation is sometimes accompanied by an increase in the activities of DNMT and HDAC [29]. Thus, ZEB1/231 cells were treated with the demethylating agent 5-aza-2'-deoxycytidine (AZA) and the HDAC inhibitor trichostatin A (TSA). These treatments significantly abolished ZEB1-mediated downregulation of *Ngn3* at the mRNA and protein levels (Figure 2E and 2F). We also examined ZEB1-regulated promoter methylation of *Ngn3* in SUM-159 cells and obtained similar results (Supplementary Figure 6).

### ZEB1 represses Ngn3 transcription by recruiting HDAC1 and DNMT3B to its promoter

**Figure 3:**
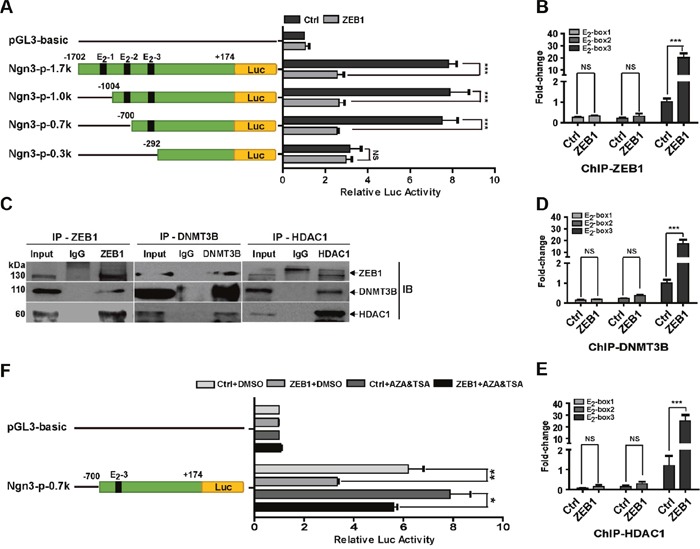
ZEB1 represses *Ngn3* transcription via interaction with DNMT3B and HDAC1 **(A)** MDA-MB-231 cells were co-transfected with the ZEB1 expression plasmid and different wild-type or truncated *Ngn3* promoter luciferase reporter constructs. Cell extract luciferase activities were determined 36 h after transfection using a Betascope analyzer. Luciferase values were normalized to Renilla activities. ***P < 0.001 vs. the respective control by Student's *t*-test. **(B)** The overexpression of ZEB1 significantly enhanced its recruitment to the endogenous *Ngn3* promoter, as confirmed by a quantitative ChIP assay using E_2_-box-specific primers. ***P < 0.001 vs. the respective control by Student's *t*-test. **(C)** The interactions among ZEB1, DNMT3B, and HDAC1 protein were analyzed by co-immunoprecipitation in ZEB1/231 cells. **(D)** and **(E)** ZEB1 overexpression significantly enhanced the recruitment of DNMT3B (D) and HDAC1 (E) to the endogenous *Ngn3* promoter, as confirmed by a quantitative ChIP assay using E_2_-box-specific primers. ***P < 0.001 vs. the respective control by Student's *t*-test. **(F)** MDA-MB-231 cells were co-transfected with the *Ngn3*-p-0.7k reporter and the ZEB1 expression plasmid, and then treated with AZA and TSA. Cell extract luciferase activities were determined 36 h after transfection using a Betascope analyzer. Luciferase values were normalized to Renilla activities. *P < 0.05, **P < 0.01 vs. the respective control by Student's *t*-test.

Next, we performed promoter-reporter assays to elucidate the molecular mechanism by which ZEB1 regulates *Ngn3* transcription. As shown in Figure 3A, the wild-type -1702/+174 promoter of the *Ngn3* gene has three canonical E_2_-box elements [CA(G/C)(G/C)TG] at positions -1308/-1303, -803/-798 and -517/-512, to which ZEB1 may potentially bind [30]. The luciferase assay results indicated that ZEB1 overexpression decreased the promoter activity of the *Ngn3*-p-1.7k reporter by approximately 68% relative to control MDA-MB-231 cells without ZEB1 transfection (Figure 3A). A series of truncated *Ngn3* promoter-reporter constructs were then generated for analysis. The luciferase assays showed that the deletion of the -1702/-700 fragment, containing E_2_-box-1 and -2, did not affect ZEB1-mediated repression of *Ngn3* promoter activity. However, this transcriptional repression was eliminated after simultaneous deletion of all three E_2_-box elements. Importantly, quantitative ChIP assays indicated that ZEB1 overexpression resulted in a 20-fold increase in its binding to the area of E_2_-box-3, whereas the recruitment of ZEB1 to E_2_-box-1 and -2 was less evident (Figure 3B and Supplementary Figure 7). These observations suggest a predominant role of the E_2_-box-3 element in the regulation of *Ngn3* transcription by ZEB1.

DNMT3B and HDAC1 have been shown to mediate the epigenetic regulatory functions of ZEB1 by acting as its cofactors [27]. We therefore performed co-immunoprecipitation experiments, which proved the interaction between ZEB1 and DNMT3B/HDAC1 in ZEB1/231 cells (Figure 3C). ChIP experiments further revealed that both DNMT3B and HDAC1 were recruited to the *Ngn3* promoter in an E_2_-box-3-dependent manner, and the association was further increased by ZEB1 overexpression (Figure 3D and 3E). Next, the ZEB1 expression plasmid and an *Ngn3* promoter construct containing E_2_-box-3 were introduced into MDA-MB-231 cells, followed by treatment with AZA and TSA. Luciferase assay results showed that exposure to AZA and TSA significantly attenuated ZEB1-mediated repression of *Ngn3* promoter activity (Figure 3F). These observations highlight the importance of the E_2_-box elements, especially E_2_-box-3, in the regulation of *Ngn3* expression by ZEB1 via interaction with DNMT3B and HDAC1.

### ZEB1 confers breast CSC properties by targeting Ngn3

**Figure 4:**
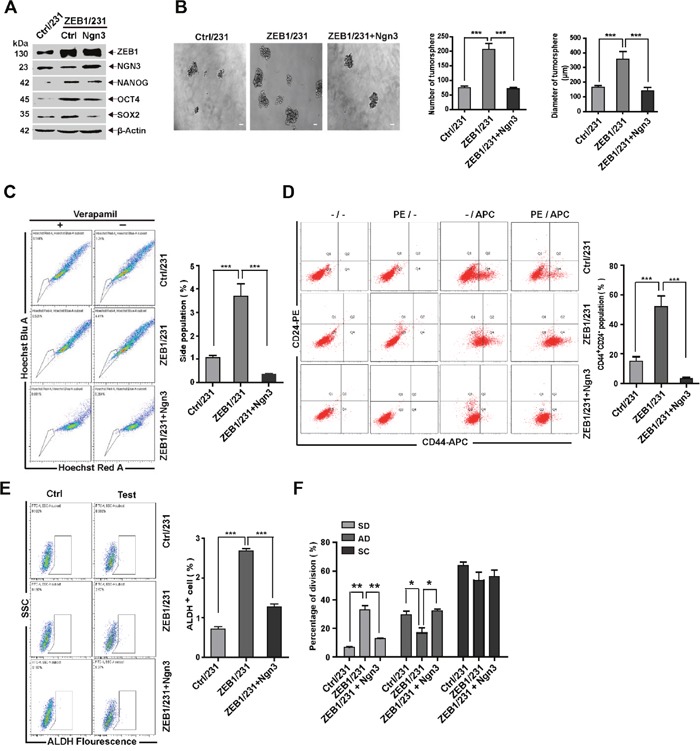
ZEB1/*Ngn3* signaling affects breast CSC properties *in vitro* **(A)** ZEB1/231 cells were transfected with an *Ngn3* expression plasmid. The expression of ZEB1, NGN3, NANOG, OCT4, and SOX2 was assessed by immunoblotting and normalized to the levels of β-Actin. **(B)** to **(F)** *Ngn3*-mediated ZEB1 regulation of CSC properties was determined by analyzing tumorsphere formation (B), side population (C), CD44^+^CD24^-^ population (D), ALDH activity (E), and SD population frequency (F). *P < 0.05, **P < 0.01, ***P < 0.001 vs. the respective control by Student's *t*-test.

Next, we tested whether ZEB1/*Ngn3* signaling would functionally alter the stemness properties of MDA-MB-231 cells. Thus, a control- or *Ngn3*-expressing plasmid was introduced into ZEB1/231 cells. Rescue of *Ngn3* expression was assessed by immunoblotting (Figure 4A). Measurements of tumorsphere formation, SP percentage, CD44^+^CD24^−^ breast CSC population, ALDH activity, and expression analysis of pluripotency genes demonstrated that ectopic ZEB1 expression led to increased stemness properties, whereas these changes were significantly attenuated by re-expression of *Ngn3* (Figure 4A-4E). Notably, as shown in Figure 4F, we observed that the SD phenotype was less represented (32.0% vs 13.3%), and the AD phenotype was enhanced (17.0% vs 31.3%), after ectopic expression of *Ngn3* in ZEB1/231 cells; this demonstrated that ZEB1 is linked to stem cell identity in breast cancer cells, thus at least partially accounting for the downregulation of *Ngn3* expression. We also examined the involvement of *Ngn3* in ZEB1-regulated cancer cell stemness properties in SUM-159 cells, with similar results (Supplementary Figure 8).

### Ngn3 is required for the induction of ZEB1-mediated CSC properties *in vivo*

**Figure 5:**
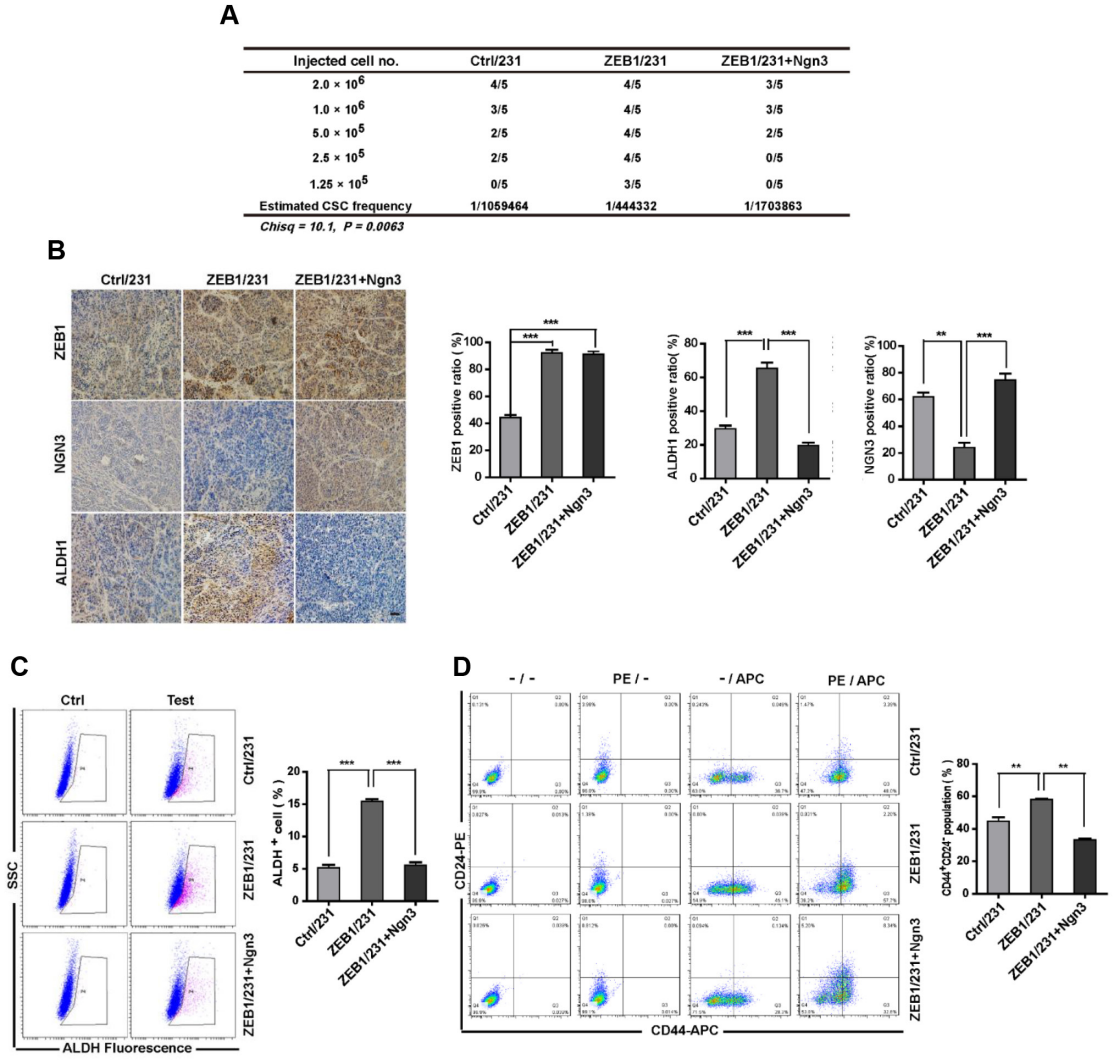
ZEB1/*Ngn3* signaling affect breast CSC properties *in vivo* **(A)** A total of 2×10^6^, 1.0×10^6^, 5×10^5^, 2.5×10^5^, and 1.25×10^5^ ZEB1/231 cells with or without *Ngn3* re-expression were injected into the mammary fat pads of nude mice. After 15 days, the mice were euthanized, and the estimated CSC frequency was analyzed using ELDA software. Data were analyzed with chi-square tests (*P = 0.0063). **(B)** The expression of ZEB1, NGN3, and ALDH1 in breast cancer xenografts was examined by immunohistochemical staining. Scale bars, 50μm. **P < 0.01, ***P < 0.001 vs respective control in Student's *t*-test. **(C)** and **(D)** Tumor tissues were prepared in single-cell suspension and processed for ALDH activity (C) and CD44^+^CD24^-^ population (D) analysis by flow cytometry. **P < 0.01, ***P < 0.001 vs. the respective control by Student's *t*-test.

Consequently, we tested whether ZEB1-regulated *Ngn3* expression would alter breast CSC frequencies *in vivo*. To do so, re-expression of *Ngn3* was performed in ZEB1/231 cells to establish a nude mouse xenograft model. The results of the extreme limiting dilution assay showed that rescuing *Ngn3* expression in ZEB1/231 tumors significantly attenuated CSC frequency (Figure 5A and Supplementary Figure 9). Immunohistochemical staining and flow cytometry analysis further revealed that the increases observed in both ALDH activity and the CD44^+^CD24^−^ CSC subpopulation in ZEB1/231 tumors were significantly abolished after re-expression of *Ngn3* (Figure 5B to 5D). Altogether, these data suggest that *Ngn3* is a ZEB1 target that may be functionally implicated in the acquisition of stem cell characteristics.

### Differential correlation of ZEB1 with NGN3 and ALDH1 expression in breast cancer specimens

**Figure 6:**
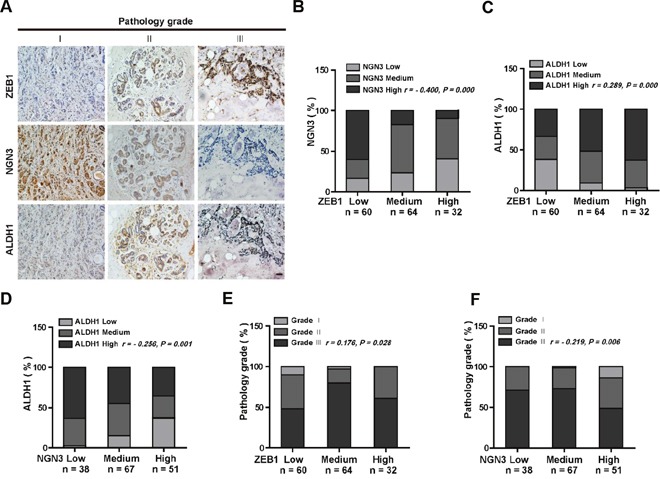
ZEB1 and NGN3 protein expression levels are inversely correlated in human breast cancer **(A)** Representative images of immunohistochemical staining of ZEB1, NGN3, and ALDH1 in serial sections of the same tumor from three cases. Scale bars, 50 μm. **(B)** The expression score for NGN3 indicates a negative correlation with ZEB1 expression in the 156 human breast cancer samples analyzed. **(C)** The expression score for ALDH1 indicates a positive correlation with ZEB1 expression. **(D)** The expression score for ALDH1 indicates a negative correlation with NGN3 expression. **(E)** The expression of ZEB1 is positively correlated with the histological grades of breast cancer. **(F)** The expression of NGN3 is negatively correlated with the histological grades of breast cancer. All data were analyzed by Spearman's rank correction test.

To further strengthen the pathological correlation between ZEB1/*Ngn3* expression and breast cancer cell stemness, we performed immunohistochemical staining for ZEB1, NGN3 and ALDH1 in 156 primary breast carcinoma patient specimens. As shown in Figure 6A, the subjects were divided into three groups on the basis of their ZEB1 expression scores. The results indicated a strong negative correlation between the expression of ZEB1 and NGN3 (Figure 6A and 6B). Importantly, in tumors with high ALDH1 activity, we observed increased expression of ZEB1 and decreased expression of NGN3 (Figure 6C and 6D), strongly suggesting that dysregulated ZEB1/NGN3 signaling is functionally linked to tumor stem cell traits in breast cancer.

We also assessed ZEB1 and NGN3 co-expression in tumors with different grades. The results revealed that ZEB1 expression was relatively lower (Figure 6E), while NGN3 expression was higher (Figure 6F) in low-grade tumors, a result consistent with findings from a previous report showing that cancer stem cells are enriched in high-grade tumors [25].

## DISCUSSION

The presence of radiation- and chemotherapy-resistant breast CSC populations contributing to tumor growth by promoting angiogenesis, tissue invasion, and metastasis is supported by multiple evidence. Therefore, the elucidation of molecular regulators of breast CSCs may translate into improved anti-neoplastic therapies. Considering our findings, we propose that ZEB1 is a key regulator of breast CSCs and may be critically involved in tumor initiation. First, we showed that ZEB1 is required for tumor initiation and expression of stemness markers *in vitro* and *in vivo*, and this effect is *Ngn3*-dependent. Second, we found that ZEB1 interacts with DNMT3B and HDAC1 at the *Ngn3* promoter, thus leading to DNA hypermethylation and downregulation of *Ngn3* expression. Third, data from *in vitro* and *in vivo* models revealed that ZEB1/*Ngn3* signaling is critical for CSC pool expansion by enhancing the frequency of the SD population. Finally, we show that the expression of ZEB1 is increased in samples from breast cancer patients and is inversely correlated with NGN3 protein levels. Therefore, our results indicated that ZEB1/*Ngn3* signaling may determine the replenishment of the breast CSC pool and promote tumor initiation.

Although ZEB1 is a well-characterized EMT regulator, several pieces of evidence suggest that it also contributes to the acquisition of CSC properties via a combination of genetic, epigenetic, and transcriptional mechanisms [31]. For example, ZEB1 interacts with HDAC components at the E-cadherin promoter in either a miR-200-dependent [32] or miR-200-independent manner [27], thus leading to induction of a mesenchymal-like phenotype and to increased tumorigenic potential. These findings suggest that ZEB1 might control tumor stemness by influencing the EMT process. However, Zhang *et al*. have reported that ZEB1 affects CSC-associated properties, such as radioresistance, independently of its ability to induce the EMT program, thus suggesting that adoption of a mesenchymal phenotype is not necessarily related to tumor onset [20]. In the present study, an essential role for ZEB1 in tumor initiation and maintenance *in vitro* and *in vivo* was confirmed by deleting ZEB1 in pre-existing breast cancer cells (MDA-MB-231 and SUM-159). Together, these findings demonstrate that ZEB1 may exert different tumorigenic functions (i.e., stemness maintenance and EMT) that are not necessarily interrelated.

Past studies of ZEB1 transcriptional activity were mostly focused on its impact within the EMT gene network, such as downregulation of epithelial cell markers (E-cadherin, PATJ, Crumbs3) and of some miR-200 family members that control epithelial-mesenchymal specification, and upregulation of mesenchymal markers (v.g. vimentin, N-cadherin, and matrix metalloproteinases [33]. On the other hand, ZEB1 has been shown to affect stemness features indirectly, through transcriptional repression of miRNAs such as miR-200 and miR-203 [18]. Here, we identified *Ngn3* as a direct ZEB1 target that mediates its effects on CSC maintenance and replenishment. NGN3 is a basic helix-loop-helix transcription factor required for controlling cell fate differentiation in multipotent progenitor cells during embryogenesis. For example, during steady state spermatogenesis, *Ngn3* expression marks a transition from strong spermatogonial stem cell potential to committed transit-amplifying cells that normally proliferate and differentiate [34]. *Ngn3* also acts as a genetic switch in pluripotent pancreatic progenitor cells, by specifying an endocrine cell fate [35]. The importance of *Ngn3* in the control of endocrine cell differentiation can also be observed in the stomach and intestine [36]. Notably, Lee *et al*. have reported that *Ngn3*^-/-^ mice display intestinal metaplasia (IM) of the gastric epithelium [37]. Given that IM is an important risk factor for gastric cancer and an inevitable stage in gastrocarcinogenesis [38], these observations suggest an inhibitory effect of *Ngn3* on the appearance of CSC characteristics that is in agreement with our data from *in vitro* and *in vivo* breast cancer models (Supplementary Figure 10 and 11). Thus, downregulation of *Ngn3* expression is at least partially involved in the mechanism by which ZEB1 promotes breast cancer cell stemness and tumor initiation and progression.

Although mainly recognized by its silencing effects on epithelial gene expression, ZEB1 can also mediate direct transcriptional activation of target genes, depending on the nature of the co-factors recruited [39, 40]. Gene silencing has been shown to occur during ZEB1 complexation with PC2-CtBP-LSD1-LCoR, or with the chromatin-remodeling protein BRG [41]; recruitment of Smad3-p300-P/CAF, in contrast, results in transcriptional activation [42]. Here we provide an alternative mechanism for ZEB1/*Ngn3*–mediated stemness acquisition in breast cancer cells that involves methylation and deacetylation of the *Ngn3* promoter. Namely, upon ZEB1 interaction with DNMT3B and HDAC1, the ensuing promoter hypermethylation and histone deacetylation result in repression of *Ngn3* expression, an effect readily reversed by inhibiting DNA methylation with AZA and histone deacetylation with TSA. Accordingly, by examining 156 primary breast cancer specimens, we confirmed that ZEB1 is overexpressed in cancer tissues, and its expression is inversely correlated with that of NGN3. Thus, dysregulation of *Ngn3* may provide a mechanistic link between the ectopic expression of ZEB1 and the ontogeny of aggressive breast cancer. These results were further supported by our analysis of cell division variants (symmetric versus asymmetric) in CSCs, which evidenced the critical role of ZEB1/*Ngn3* signaling in replenishing the breast CSC pool and promoting tumorigenesis. Similarly to our own conclusions regarding ZEB1, the polarity of cell division has been shown to be affected by p53 in murine mammary stem cells; in this model, p53 loss leads to uniform NUMB redistribution in the stem cell undergoing mitosis, enhances mammospheres’ replication potential, and increases the rate of SD [43]. Although in such study the underlying mechanism remained undefined, a recent study implicated ZEB1 as a direct transcriptional target of p53 [44]; thus we believe that ZEB1 signaling may be involved.

In summary, our study uncovered a key role for ZEB1 in the establishment of CSC properties and in promoting tumor initiation by breast CSCs. Since ZEB1 may affect different steps in the tumorigenic process, more detailed studies are needed to elucidate its possible contribution to tumor initiation and expansion. In addition, given that ZEB1 downregulation parallels metastatic colonization [45, 46], its possible contribution to the metastatic process should also be addressed.

## MATERIALS AND METHODS

### Cell culture and transfection

Human breast cancer cell lines were maintained in DMEM (SUM-159) and RPMI 1640 (MDA-MB-231) supplemented with 10% FBS, 100 IU penicillin, and 100 mg/mL streptomycin. Cells were transfected using Lipofectamine 2000 (Invitrogen, California, USA) according to the manufacturer's protocol.

### Plasmid construction

The human cDNA fragment encoding full-length *ZEB1* and *Ngn3* was prepared by PCR and cloned into pLV-EF1-MCS-IRES-Bsd (Biosettia, San Diego, USA). The lentiviral-based vector pLV-H1-EF1α-puro (Biosettia, San Diego, USA) was used to express shRNAs in breast cancer cells. The human *Ngn3* full-length and truncated promoter sequences were obtained by PCR from human genomic DNA and cloned into the pGL3-basic vector (Promega, Wisconsin, USA). Primer sequences are listed in Supplementary Data.

### Generation of lentiviruses

Lentiviruses were generated by transfecting subconfluent HEK293T cells with lentiviral vectors and packaging plasmids via calcium phosphate transfection. Viral supernatants were collected 48 h after transfection, resuspended, and filtered through 0.45 μm filters (Millipore, Massachusetts, USA). MDA-MB-231 or SUM-159 cells were transduced with lentivirus-containing medium and selected with antibiotic. After one week, cell pools were obtained and expanded.

### RNA extraction and quantitative RT-PCR

Total RNA (0.5 μg) from each sample was collected using TRIzol reagent (Invitrogen, California, USA), and first-strand cDNA synthesis was performed using M-MLV Reverse Transcriptase (Takara, Tokyo, Japan). The specific products were amplified by quantitative PCR using a TransStart Green Q-PCR SuperMix Kit (TransGen, Beijing, China). GAPDH was used as a normalization control. Primer sequences are listed in Supplementary Data.

### Luciferase assay

Cells were co-transfected with full-length or truncated human *Ngn3* promoters and ZEB1 expression plasmid in 24-well plates. Lysates were prepared 36 h after transfection, and luciferase activity was measured using the Dual-Luciferase Reporter Assay System (Promega, Wisconsin, USA) according to the manufacturer's protocol. Luciferase activity was normalized to Renilla luciferase activity.

### Immunoblotting assay

The preparation of total cell extracts and immunoblotting with appropriate antibodies (indicated in Supplementary Data) were performed as previously described [47]. Labeled proteins were visualized with an ECL chemiluminescence kit (Millipore, Massachusetts, USA).

### Tumorsphere assay

Single-cell suspensions were prepared in sphere-culturing medium (Stemcell Technologies) and seeded in 24-well ultra-low attachment plates (Corning) at 1,000 cells/well. The number of wells with spheres was recorded on day 14 after culturing. Tumorsphere formation was monitored using an inverted Leica microscope fitted with a camera. Sphere numbers and diameters were imaged and quantified in >5 fields per sample.

### Flow cytometry

The ALDEFLUOR assay (Stemcell Technologies) was performed according to the manufacturer's guidelines to identify cells with high ALDH activity. Cells were incubated for 40 minutes at 37 °C in the presence or absence of the ALDH inhibitor diethylaminobenzaldehyde. To identify the CD44^+^CD24^-^ population, CD44-APC and CD24-PE primary antibodies (BD, USA) were incubated with single cells in PBS containing 2% FBS for 30 minutes at 4°C. Then, cells were washed twice with PBS, resuspended in 1 mL PBS, and kept on ice until FACS analysis. To identify the side population (SP) and non-side population fractions, cells were trypsinized and suspended at a concentration of 1×10^6^ cells/mL in PBS containing 2% FBS. Hoechst 33342 dye (Sigma-Aldrich, Vienna, Austria) was added at a final concentration of 5 μg/mL in the presence or absence of 100 μM verapamil (Sigma-Aldrich), and the cells were incubated at 37°C for 90 min with intermittent shaking every 15 min. The cells were washed twice with PBS and resuspended in 1 mL PBS with 1 μg/mL propidium iodide and kept on ice until FACS analysis. Cell analyses were carried out with a FACSAria instrument (BD, USA).

### Methylation assays

DNA was extracted from breast cancer cells and processed for bisulfite treatment (Qiagen, Hilden, Germany). Bisulfite-treated DNA was then used to examine the methylation status of the CpG islands in the *Ngn3* promoter, using Bisulfite-Sequencing PCR (BSP) according to the manufacturer's protocol (Roche, Basel, Switzerland). Primer sequences are listed in Supplementary Data.

### Immunoprecipitation assay

Cell lysates were incubated with specific antibodies and Protein G agarose beads (Invitrogen, California, USA) at 4°C overnight, then washed three times with a buffer containing 50 mM Tris (pH 7.5), 100 mM NaCl, 7.5 mM EGTA, and 0.1% Triton X-100. The antibodies used for immunoprecipitation are shown in Supplementary Data.

### Chromatin immunoprecipitation

ChIP assays were performed using an EZ-ChIP kit (Millipore, Massachusetts, USA) according to the manufacturer's instructions. The antibodies used in these experiments are shown in Supplementary Data. The fragments of human *Ngn3* promoter containing the E_2_-box 1, E_2_-box 2 and E_2_-box 3 elements in immunoprecipitates were amplified by quantitative PCR. Primer sequences are listed in Supplementary Data.

### Tissue samples

A total of 156 human breast cancer specimens were obtained from the General Hospital of the People's Liberation Army (PLAGH, Beijing, China). All patients had histologically confirmed invasive ductal carcinoma of the breast and were recruited by the same department. This study was approved by the institutional ethics committees at PLAGH and the Medical College of Nankai University.

### Immunohistochemical analysis

Immunohistochemical analysis of paraffin-embedded sections was performed using an Envision Kit (Dako, Denmark) according to the manufacturer's protocol. Sections were boiled in retrieval solution to expose antigens. Specific antibodies (see Supplementary Data) were applied to the sections. Slides were counterstained with hematoxylin, dehydrated, and mounted. Immunostaining was independently evaluated by 2 pathologists.

### Cell division assay

CD44^+^CD24^-^ cells were treated with 20 mM blebbistatin (Selleck Chemicals, USA) for 48 hours in suspension culture and fixed for 20 minutes in 10% neutral buffered formalin at -20°C. Fixed cells were blocked for 1.5 hours with 5% goat serum and then incubated with anti-Numb and anti-CD44 primary antibodies diluted in TBST (containing 0.1% Tween 20 and 5% BSA) overnight at 4°C. Then, cells were washed 3 times with TBST at RT, incubated with secondary antibodies for 1 hour, then washed 3 times at RT. The secondary antibodies were DyLight 488 goat anti-mouse and DyLight 594 goat anti-rabbit. Cell nuclei were counterstained and mounted with Prolong Gold Antifade Reagent with DAPI (Molecular Probes) overnight at RT.

### Tumor xenograft experiments

All experimental procedures involving animals were performed according to the institutional ethical guidelines for animal experiments and approved by the Ethics Committee for Animal Use at the Medical College of Nankai University. Briefly, cells were collected and suspended at different concentrations (1.0×10^7^, 5.0×10^6^, 2.5×10^6^, 1.25×10^6^, and 6.25×10^5^ cells/mL) in 200 μL PBS, and then injected into the mammary fat pads of female BALB/c nude mice. After 15 days, mice were euthanized, and the tumor tissues were processed for detection of the CD44^+^CD24^-^ population and ALDH activity and sectioned for histological evaluation. For limited dilution assays, data analysis was performed using the publicly available ELDA (extreme limiting dilution analysis for comparing depleted and enriched populations in stem cell and other assays) software [48].

### Isolation of primary tumor cells

Tumors were surgically removed from anesthetized mice and minced with a scalpel in RPMI medium containing trypsin (1 mg/mL) and collagenase I (3 mg/mL); 2% FBS was added, and the tissue fragments were incubated for 45 minutes in a shaker (37°C, 90 rpm). The dissociated cells were filtered through 40 μm cell strainers (BD, USA) and concentrated by centrifugation at 1,500 rpm for 15 minutes at RT. Cells were washed with 50 mL volumes of PBS until the supernatant was clear of red blood cells.

### Statistical analysis

Statistical analyses were performed using SPSS 13.0 software (SPSS, Chicago, IL, USA). Data are presented as the means ± SD and represent three independent experiments. Spearman's rank correlation test was used to analyze the correlation of gene expression in tissue samples. One-way analysis of variance (ANOVA) was used to compare means between treatment groups. Where appropriate, Student's *t*-test for unpaired observations was applied. A *P*-value < 0.05 was considered significant.

## SUPPLEMENTARY MATERIALS FIGURES AND TABLES


