# Supplementary material for: ZEB1 confers stem cell-like properties in breast cancer by targeting neurogenin-3
Source: Oncotarget. 2017 Apr 13;8(33):54388–401. doi: 10.18632/oncotarget.17077 (PMC5589589; doi:10.18632/oncotarget.17077)
Supplement: Supplementary file 1 [file oncotarget-08-54388-s001.pdf]

ZEB1 confers stem cell-like properties in breast cancer by targeting neurogenin-3

SUPPLEMENTARY MATERIALS AND METHODS

Primers

Construction of full-length ZEB1 expression vector	
forward	5’-ATGGCGGATGGCCGCC-3’
reverse	5’-TTAGGCTTCATTTGTCTTTTCTTCA-3’
Construction of full-length *Ngn3* expression vector	
forward	5’-TGCTCTAGAATGACGCCTCAACCC-3’
reverse	5’-CGCGGATCCGCGACAGACAGGTCCT-3’
Construction of shRNA vector	
shZEB1-1	5’-CGGCGCAATAACGTTACAAAT-3’
shZEB1-2	5’-GGCGCAATAACGTTACAAA-3’
shZEB1-3	5’-CCTCTCTGAAAGAACACATTA-3’
sh*Ngn3*-1	5’-AGCGCAATCGAATGCACAACC-3’
sh*Ngn3*-2	5’-GCGCAATCGAATGCACAACCT-3’
sh*Ngn3*-3	5’-GCAATCGAATGCACAACCTCA-3’
Construction of *Ngn3* promoter	
-292-forward	5’- TTGGAGCAAGGCAAGGGGA-3’
-700-forward	5’- AGCAATCCCTAAATAATCTAATA-3’
-1004-forward	5’- TCAGTTGTCCTAGTTGGAGGCTTGT-3’
-1702-forward	5’- AAGACACCACCTTTGCACTCTAATG-3’
Reverse	5’- AAAGGACCTGTCTGTCGCTG-3’
Bisulfite sequencing PCR	
BSP forward	5’-TTGGGGAGATGTTTTTTGGT-3’
BSP reverse	5’-TCAATCCAATATTTAATCTCTCAAC-3’
Quantitative RT-PCR	
human ZEB1 forward	5’-CAGCTTGATACCTGTGAATGGG-3’
human ZEB1 reverse	5’-TATCTGTGGTCGTGTGGGACT-3’
human *Ngn3* forward	5’-CTAAGAGCGAGTTGGCACTGA-3’
human *Ngn3* reverse	5’-GAGGTTGTGCATTCGATTGCG-3’
Quantitative ChIP	
E_2-box-1 forward	5’- TGCTCATGCTGCACCAGTC-3’
E_2-box-1 reverse	5’- AAGTGCTGTGTCGGTGGAGTCA-3’
E_2-box-2 forward	5’- GTCCTAGTTGGAGGCTTG-3’
E_2-box-2 reverse	5’- TGGCAAGCTAATGTTATT-3’
E_2-box-3 forward	5’- ACAAATGTGTAAAACAGG-3’
E_2-box-3 reverse	5’- GGCGGAAGACCAGGCTG -3’

Antibodies

Marker	Species	Application	Manufacturer	Catalog No.	Dilution
anti-ZEB1	Rabbit	IHC	Abcam	ab87280	1:100
	Rabbit	IB	Santa Cruz	sc-25388	1:1000
	Rabbit	IP	Proteintech	221544-1-AP	2 μg/reaction
		CHIP			1 μg/reaction
anti-NGN3	Rabbit	IHC	Abcam	ab38548	1:100
		IB			1:1000
anti-ALDH	Rabbit	IHC	Abcam	ab52492	1:100
anti-CD44-APC	Mouse	F	BD	559942	1:100
anti-CD24-PE	Mouse	F	BD	555428	1:100
anti-CD44	Rabbit	IF	Abcam	ab51037	1:100
anti-SOX2	Rabbit	IB	CST	#3579	1:1000
anti-OCT4	Rabbit	IB	CST	#2750	1:1000
anti-NANOG	Rabbit	IB	CST	#4903	1:1000
anti-NUMB	Mouse	IF	Abcam	ab13891	1:100
anti-DNMT3B	Mouse	IP	Abcam	ab13604	2 μg/reaction
		CHIP			1 μg/reaction
anti-HDAC1	Rabbit	IP	Abcam	ab58164	2 μg/reaction
		CHIP			1 μg/reaction
anti-β-Actin	Mouse	IB	Sigma	A-4700	1:5000

IHC: Immunohistochemistry; IB: Immunoblotting; IF: Immunofluorescence; IP: Immunoprecipitation; ChIP: Chromatin immunoprecipitation; F: Flow cytometry.

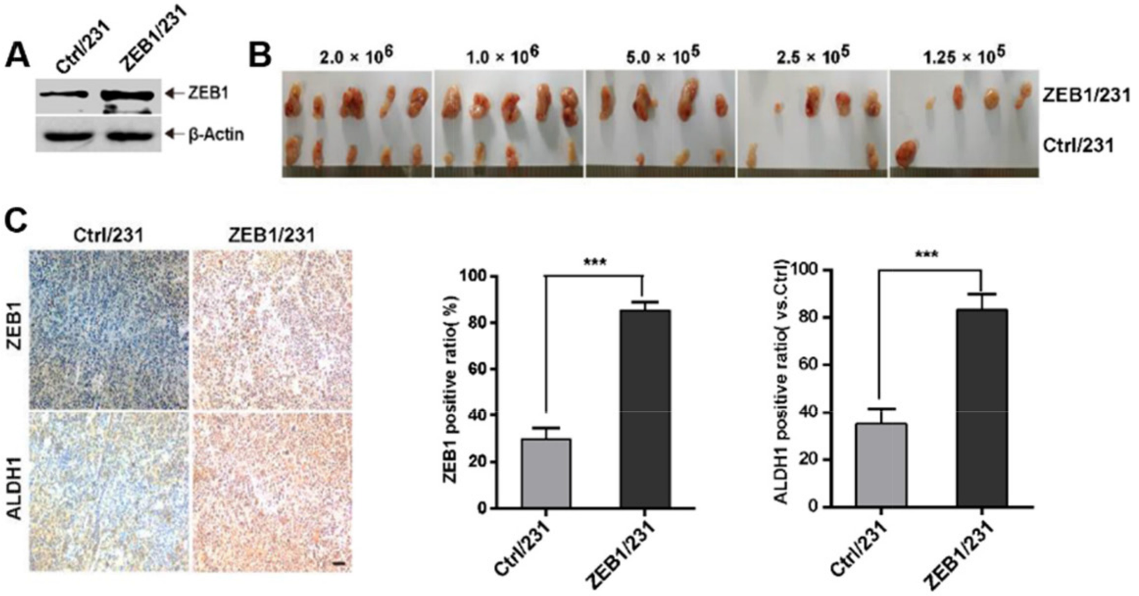

Supplementary Figure 1: ZEB1 increases tumor initiation in MDA-MB-231 xenograft *in vivo*. **(A)** MDA-MB-231 cells were stably transfected with the ZEB1 expression plasmid (ZEB1/231) or empty vector control (Ctrl/231). The expression of ZEB1 was verified by immunoblotting and normalized to the levels of β-Actin. **(B)** For *in vivo* limited dilution assays, a total of 2×10^6, 1.0×10^6, 5×10^5, 2.5×10^5, and 1.25×10^5 ZEB1/231 or Ctrl/231 cells were injected into the mammary fat pads of nude mice. After 15 days, the mice were euthanized. Tumors from mice in each group were shown. **(C)** The expression of ZEB1 and ALDH1 in breast cancer xenograft tumors was examined by immunohistochemical staining. ***$P < 0.001$ vs. the respective control by Student's t-test. Scale bars, 20 µm.

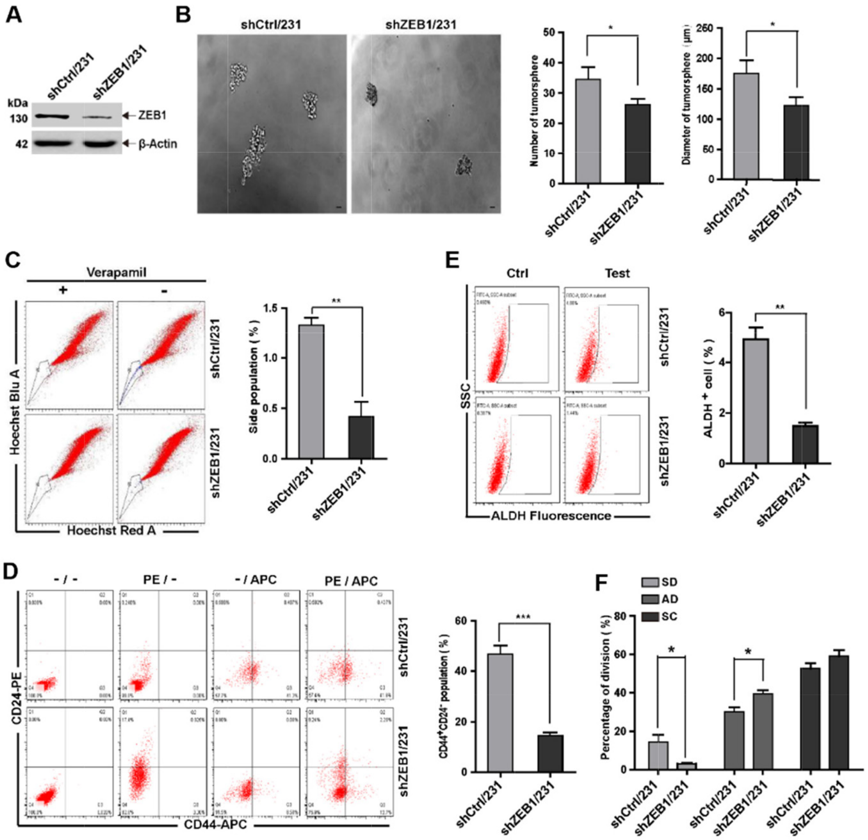

Supplementary Figure 2: Knockdown of ZEB1 attenuates MDA-MB-231 stemness properties *in vitro*. **(A)** MDA-MB-231 cells were stably transfected with the specific shRNA targeting ZEB1. The expression of ZEB1 was verified by immunoblotting and normalized to the levels of β-Actin. **(B)** to **(F)** ZEB1-regulated stemness properties was determined by tumorsphere formation (B), side population (C), $CD44^{+}CD24^{-}$ population (D), ALDH activity (E), and the frequency of SD population (F) analysis. *$P < 0.05$, **$P < 0.01$, ***$P < 0.001$ vs. the respective control by Student's *t*-test.

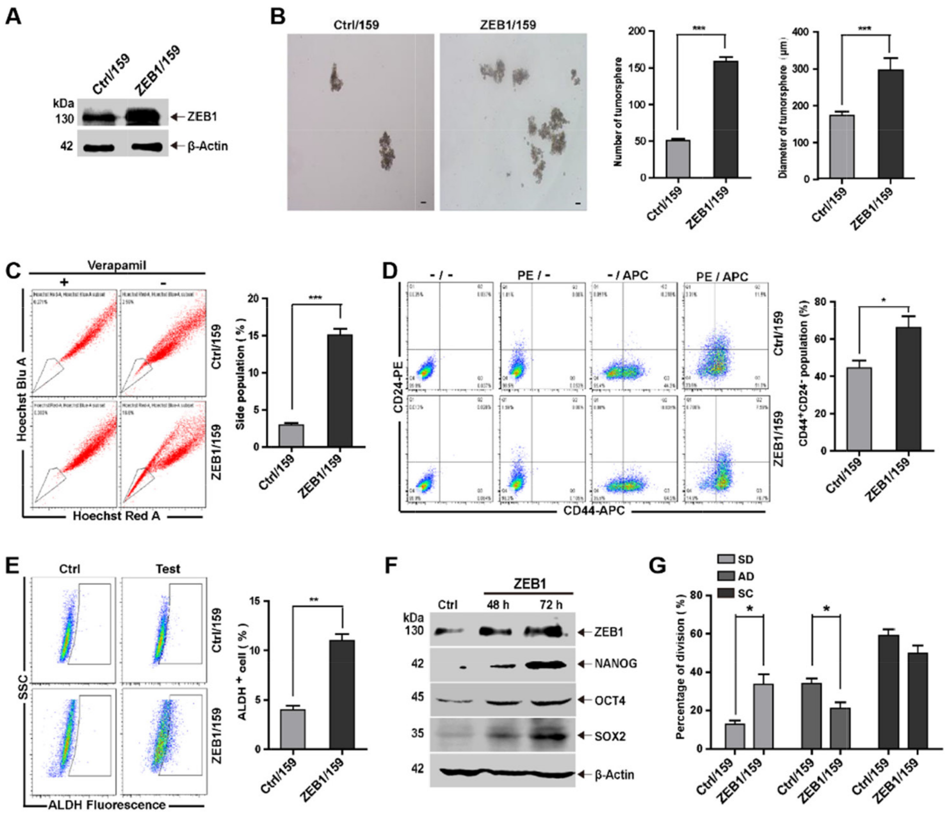

Supplementary Figure 3: Ectopic ZEB1 increases SUM-159 stemness properties *in vitro*. **(A)** SUM-159 cells were stably transfected with the ZEB1 expression plasmid. The expression of ZEB1 was verified by immunoblotting and normalized to the levels of β-Actin. **(B)** to **(G)** ZEB1-regulated stemness properties was determined by tumorsphere formation (B), side population (C), $CD44^{+}CD24^{-}$ population (D), ALDH activity (E), stemness-related genes (F), and the frequency of SD population (G) analysis. *P < 0.05, **P < 0.01, ***P < 0.001 vs. the respective control by Student's t-test.

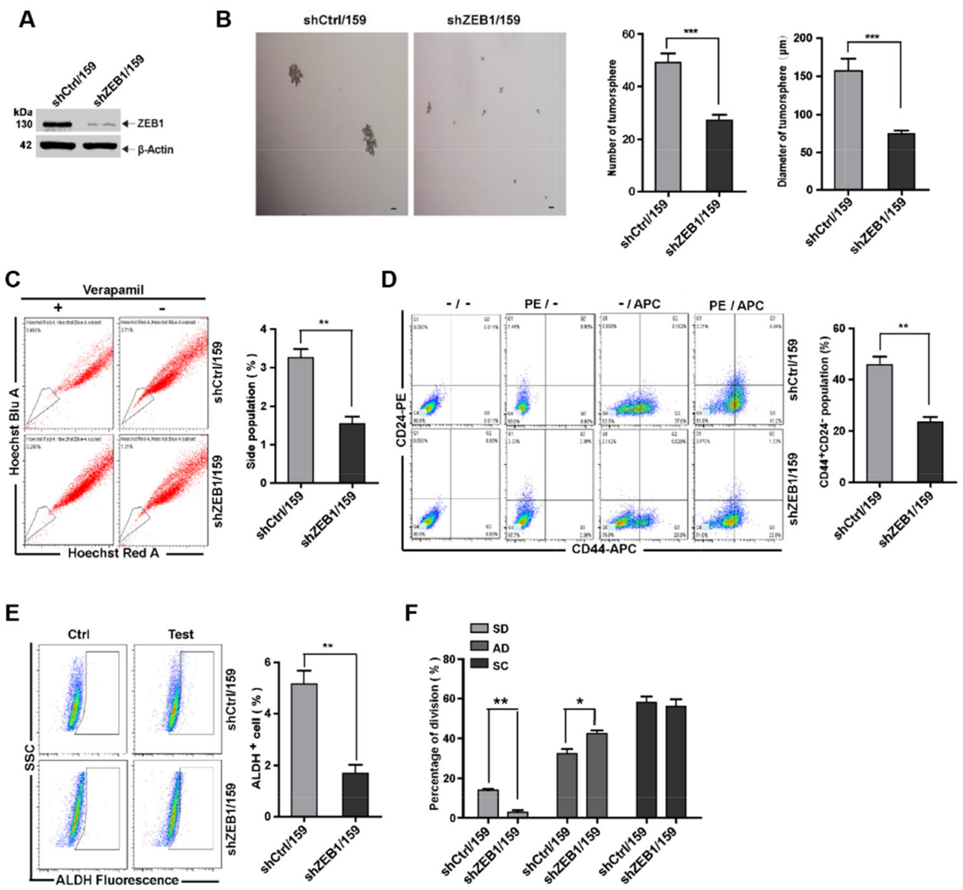

Supplementary Figure 4: Knockdown of ZEB1 attenuates SUM-159 stemness properties *in vitro*. (A) SUM-159 cells were stably transfected with the specific shRNA targeting ZEB1. The expression of ZEB1 was verified by immunoblotting and normalized to the levels of β-Actin. **(B)** to **(F)** ZEB1-regulated stemness properties was determined by tumorsphere formation (B), side population (C), $CD44^{+}CD24^{-}$ population (D), ALDH activity (E), and the frequency of SD population (F) analysis. $^{*}P < 0.05$, $^{**}P < 0.01$, $^{***}P < 0.001$ vs. the respective control by Student's *t*-test.

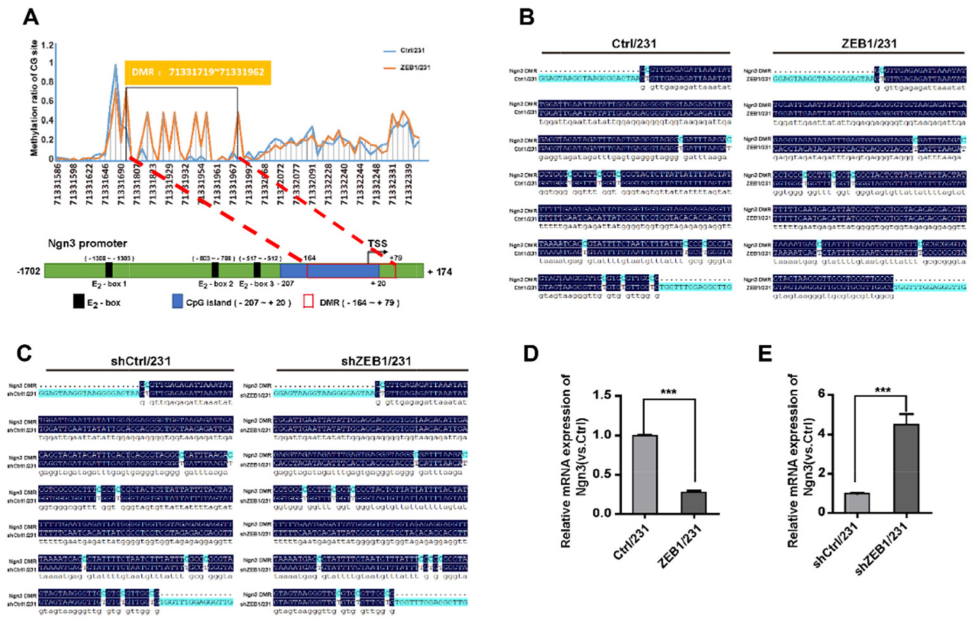

Supplementary Figure 5: ZEB1 regulates DNA methylation of the *Ngn3* promoter in MDA-MB-231 cells. **(A)** RRBS analysis shows that ectopic ZEB1 increases the methylation ratio of DMRs located in the *Ngn3* promoter. The DMRs region, the CpG island and three canonical E_2-box elements for ZEB1 binding were identified within the *Ngn3* promoter. **(B)** Overexpression of ZEB1 results in hypermethylation of the *Ngn3* promoter by BSP analysis. **(C)** Downregulation of ZEB1 results in hypomethylation of the *Ngn3* promoter by BSP analysis. **(D)** and **(E)** The mRNA expression of *Ngn3* was examined by quantitative PCR in ZEB1/231 vs. Ctrl/231 cells (D) and in shZEB1/231 vs. shCtrl/231 cells (E). ***$P < 0.001$, vs. the respective control by Student's *t*-test.

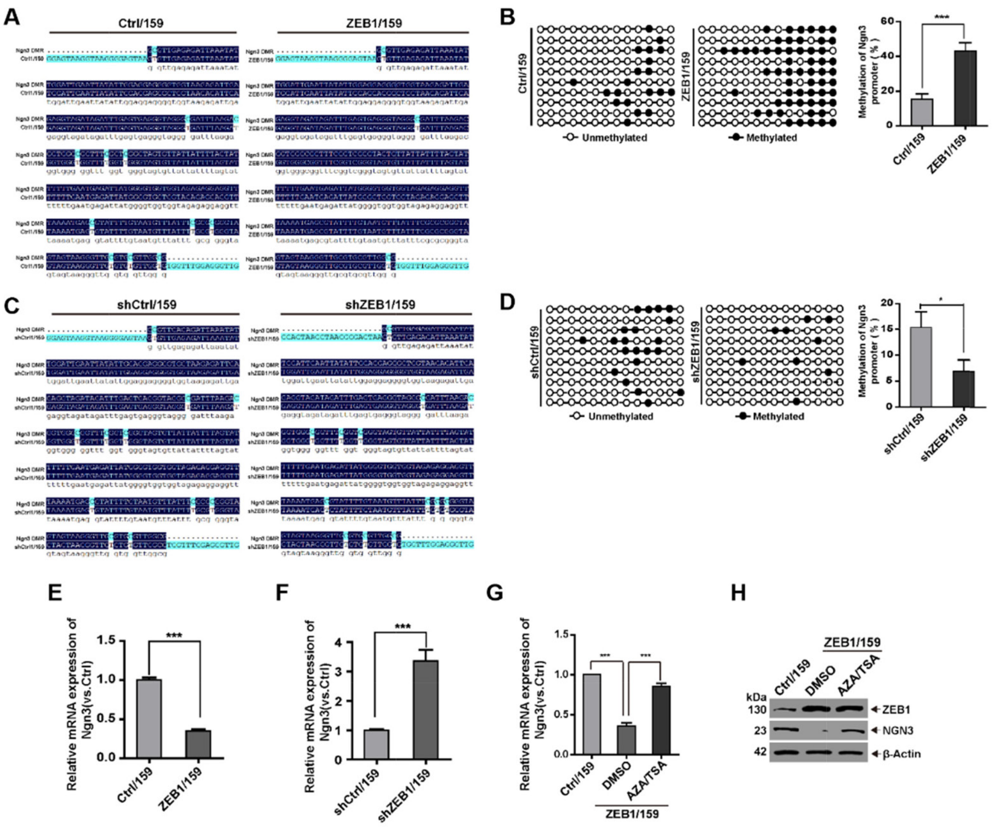

Supplementary Figure 6: ZEB1 regulates DNA methylation of the *Ngn3* promoter in SUM-159 cells. **(A)** Overexpression of ZEB1 results in hypermethylation of the *Ngn3* promoter by BSP analysis. **(B)** The percentage of DNA methylation of the *Ngn3* promoter is increased in ZEB1/159 vs. Ctrl/159 cells. ****P* < 0.001 vs. the respective control by Student's *t*-test. **(C)** Downregulation of ZEB1 results in hypomethylation of the *Ngn3* promoter by BSP analysis. **(D)** The percentage of DNA methylation of the *Ngn3* promoter is decreased in shZEB1/159 vs. shCtrl/159 cells. **P* < 0.05 vs. the respective control by Student's *t*-test. **(E)** and **(F)** The mRNA expression of *Ngn3* was examined by quantitative PCR in ZEB1/159 vs. Ctrl/159 cells (E) and in shZEB1/159 vs. shCtrl/159 cells (F). ****P* < 0.001, vs. the respective control by Student's *t*-test. **(G)** and **(H)** ZEB1/159 and Ctrl/159 cells were treated with AZA (1.5 µM) and TSA (2 mM) for the indicated time points. The expression of *Ngn3* was assessed by quantitative PCR (E) and immunoblotting (F) and normalized to the levels of β-Actin. ****P* < 0.001 vs. the respective control by Student's *t*-test.

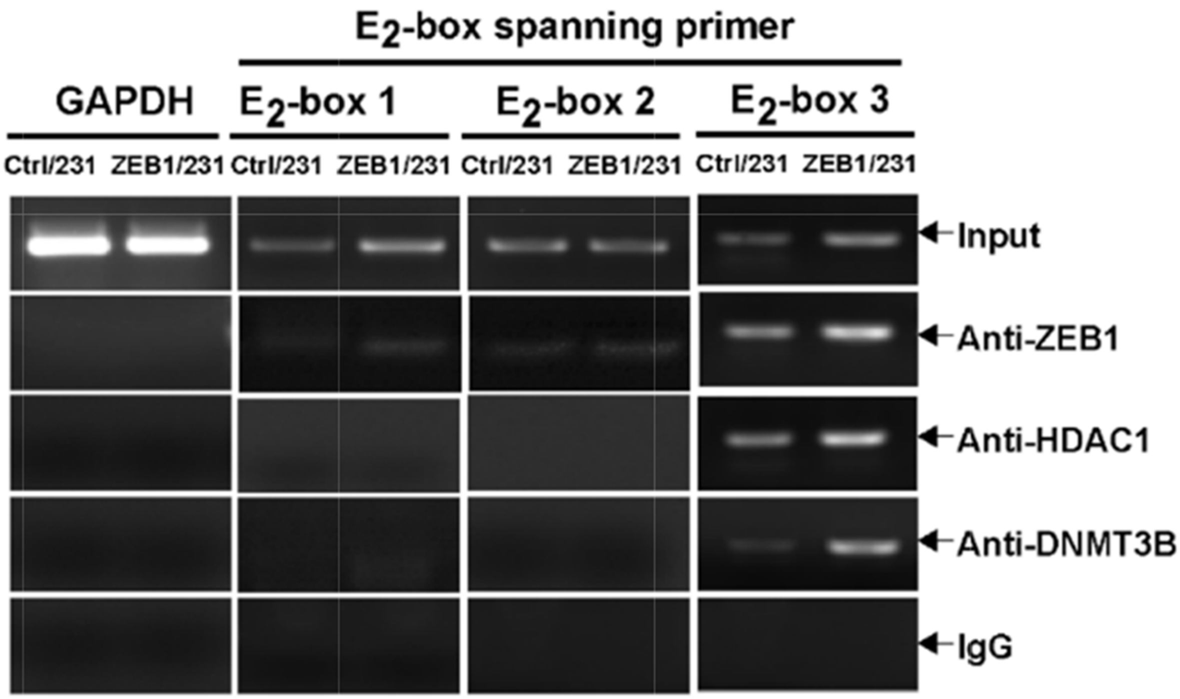

Supplementary Figure 7: ZEB1 recruits to the *Ngn3* promoter in an E_2-box dependent manner by forming a complex with HDCA1 and DNMT3B. The association of ZEB1, HDCA1 and DNMT3B with the *Ngn3* promoter was analyzed by ChIP assay. The amplified sequence of the *Ngn3* promoter fragment containing E_2-box-1 and E_2-box-2 and E_2-box-3 elements is shown. Input DNA amounts were confirmed by equal loading of chromatin.

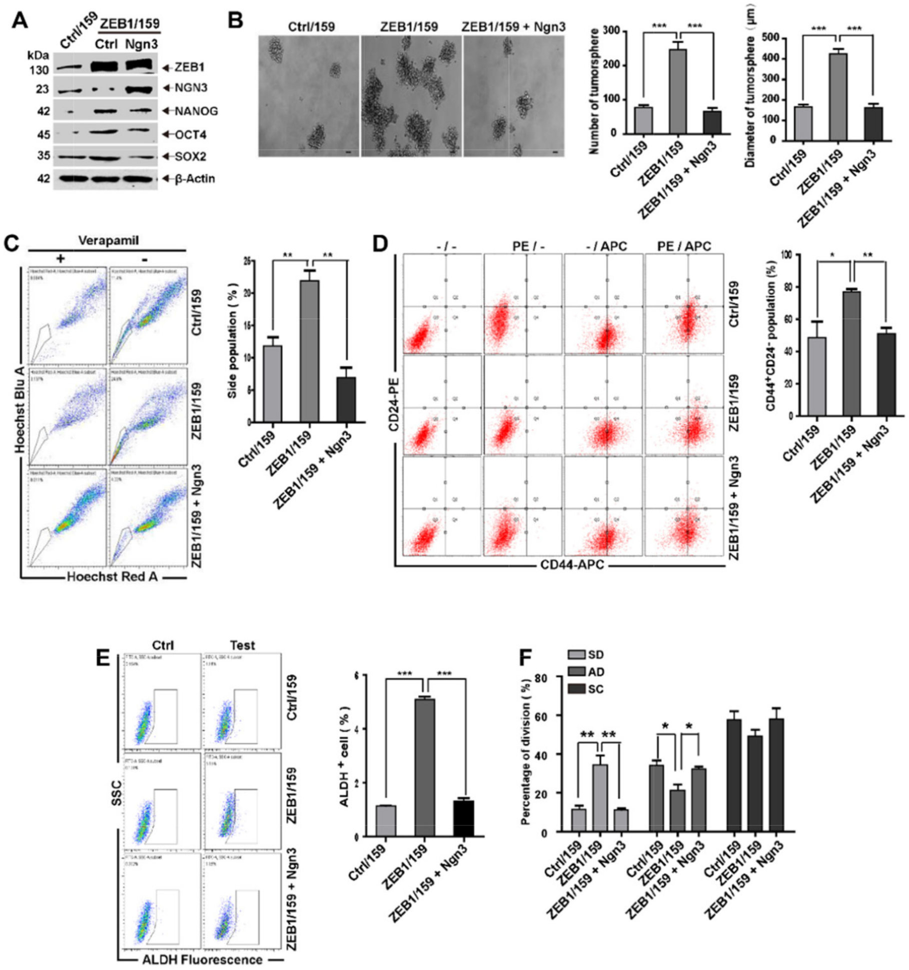

Supplementary Figure 8: ZEB1/*Ngn3* signaling is involved in breast CSC properties *in vitro* in SUM-159 cells. (A) ZEB1/159 cells were transfected with *Ngn3* expression plasmid. The expression of ZEB1, NGN3, NANOG, OCT4, and SOX2 was assessed by immunoblotting and normalized to the levels of β-Actin. **(B)** to **(F)** *Ngn3*-mediated ZEB1 regulation of CSC properties was determined by tumorsphere formation (B), side population (C), CD44$^+$CD24$^-$ population (D), ALDH activity (E), and the frequency of SD population analysis (F), $*P < 0.05$, $**P < 0.01$, $***P < 0.001$ vs. the respective control by Student's *t*-test.

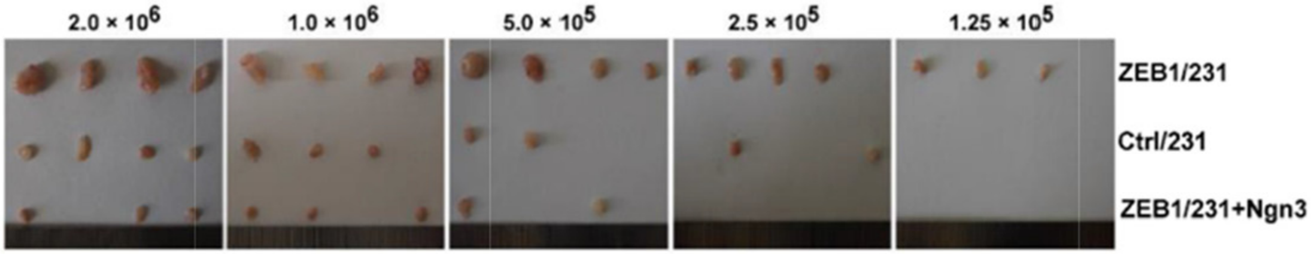

Supplementary Figure 9: ZEB1/*Ngn3* signaling is involved in tumor initiation in MDA-MB-231 xenograft *in vivo*. A total of 2×10^6, 1.0×10^6, 5×10^5, 2.5×10^5, and 1.25×10^5 ZEB1/231 cells with or without *Ngn3* re-expression were injected into the mammary fat pads of nude mice, respectively. After 15 days, the mice were euthanized. Tumors from mice in each group were shown.

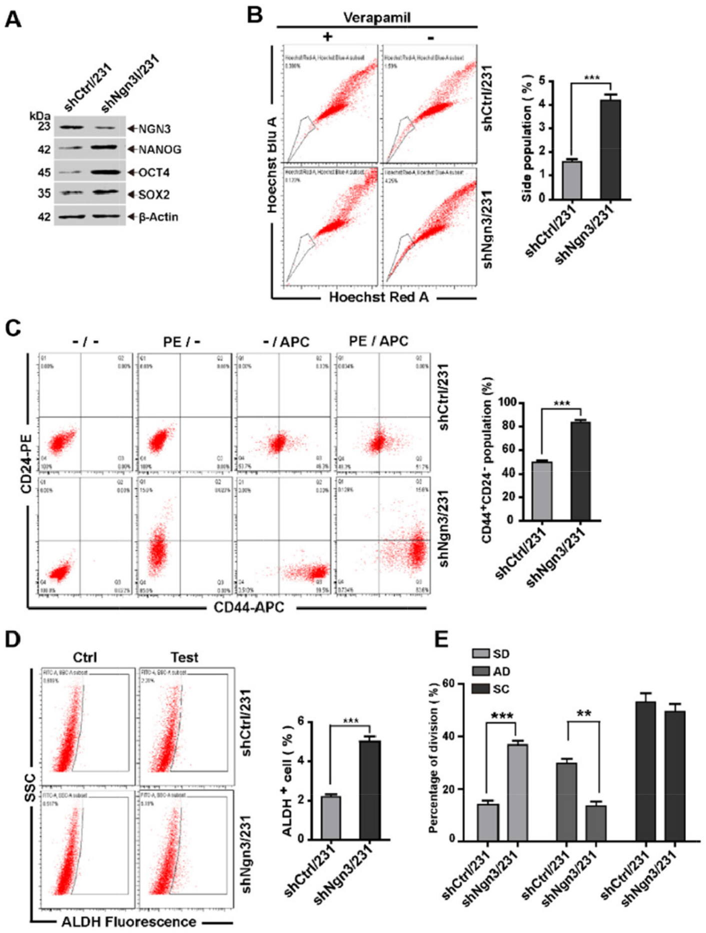

Supplementary Figure 10: Downregulation of *Ngn3* expression increases MDA-MB-231 stemness properties *in vitro*. **(A)** MDA-MB-231 cells were stably transfected with the specific shRNA targeting *Ngn3*. The expression of NGN3 and stemness-related genes was verified by immunoblotting normalized to the levels of β-Actin. **(B)** to **(E)** *Ngn3*-regulated stemness properties was determined by side population (B), $CD44^+CD24^-$ population (C), ALDH activity (D), and the frequency of SD population (E) analysis. ***P* < 0.01, ****P* < 0.001 vs. the respective control by Student's *t*-test.

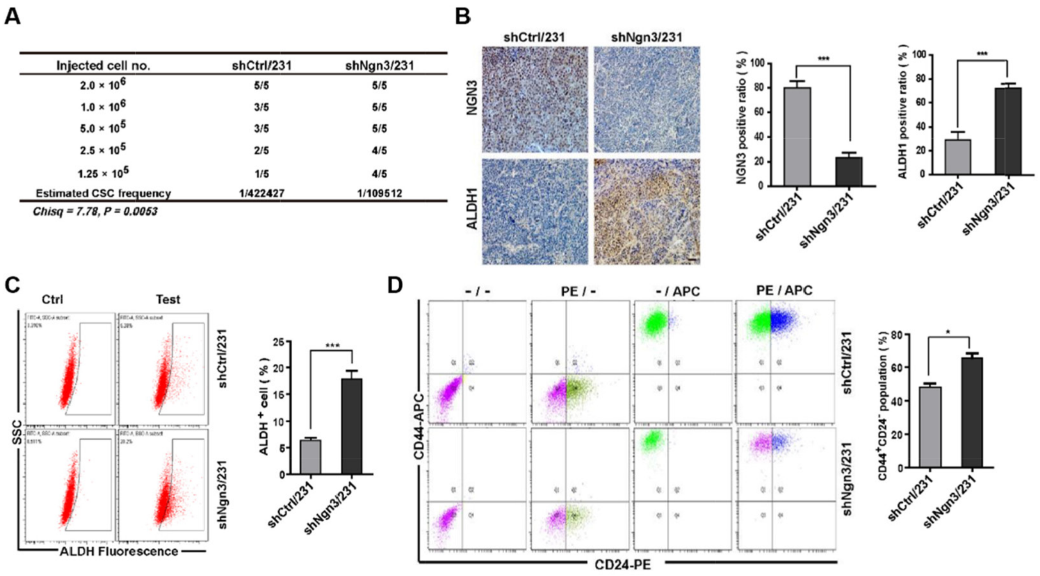

Supplementary Figure 11: Downregulation of *Ngn3* expression increases tumor initiation and stmness properties in MDA-MB-231 xenograft *in vivo*. **(A)** For *in vivo* limited dilution assay, a total of $2{\times}10^6$, $1.0{\times}10^6$, $5{\times}10^5$, $2.5{\times}10^5$, and $1.25{\times}10^5$ sh*Ngn3*/231 or shCtrl/231 cells were injected into the mammary fat pads of nude mice, respectively. After 15 days, the mice were euthanized and estimated CSC frequency was analyzed using the ELDA software. Data was analyzed by chi-square test (*P = 0.0053). **(B)** The expression of NGN3 and ALDH1 in breast cancer xenografts was examined by immunohistochemical staining. Scale bars, 50µm. ***P < 0.001 vs respective control in Student's *t*-test. **(C)** and **(D)** Tumor tissues were prepared in single-cell suspension and processed for ALDH activity (C) and $CD44^+CD24^-$ population (D) analysis by flow cytometry. *P < 0.05, ***P < 0.001 vs. the respective control by Student's *t*-test.
